# Effect of exosomes from adipose‐derived stem cells on the apoptosis of Schwann cells in peripheral nerve injury

**DOI:** 10.1111/cns.13187

**Published:** 2019-07-06

**Authors:** Cai‐Yue Liu, Gang Yin, Yi‐Dan Sun, Yao‐Fa Lin, Zheng Xie, Arthur W. English, Qing‐Feng Li, Hao‐Dong Lin

**Affiliations:** ^1^ Department of Orthopedic Surgery, Shanghai General Hospital Shanghai Jiaotong University School of Medicine Shanghai China; ^2^ Department of Plastic and Reconstructive Surgery, Shanghai Ninth People's Hospital Shanghai Jiaotong University School of Medicine Shanghai China; ^3^ Department of Plastic Surgery, Changzheng Hospital The Navy Military Medical University Shanghai China; ^4^ Department of Orthopedic Surgery, Changzheng Hospital The Navy Military Medical University Shanghai China; ^5^ Department of Cell Biology, School of Medicine Emory University Atlanta GA USA

**Keywords:** adipose‐derived stem cells, apoptosis, exosomes, peripheral nerve injury, Schwann cells

## Abstract

**Aims:**

Recovery after peripheral nerve injury (PNI) is often difficult, and there is no optimal treatment. Schwann cells (SCs) are important for peripheral nerve regeneration, so SC‐targeting treatments have gained importance. Adipose‐derived stem cells (ADSCs) and their exosomes can promote peripheral nerve repair, but their interactions with SCs are unclear.

**Methods:**

Purified SCs from sciatic nerve injury sites were harvested, and apoptosis and proliferation of SCs at post‐PNI 24 hours were analyzed. The effects of coculture with ADSCs and different concentrations of ADSC‐derived exosomes (ADSC‐Exo) were studied through in vitro experiments by flow cytometry, CCK8 assay, immunofluorescence staining, and histological analysis. The expression of the apoptosis‐related genes Bcl‐2 and Bax was also analyzed by qRT‐PCR.

**Results:**

ADSC‐Exo reduced the apoptosis of SCs after PNI by upregulating the anti‐apoptotic Bcl‐2 mRNA expression and downregulating the pro‐apoptotic Bax mRNA expression. Further, it also improved the proliferation rate of SCs. This effect was confirmed by the morphological and histological findings in PNI model rats.

**Conclusion:**

Our results present a novel exosome‐mediated mechanism for ADSC‐SC cross talk that reduces the apoptosis and promotes the proliferation of SCs and may have therapeutic potential in the future.

## 1 AIMS

Peripheral nerve injury (PNI) has been gaining increasing attention on account of its increased prevalence with the rapid development of society.^1^ The major causes of PNI, such as diabetes mellitus, autoimmune disease, infection, drug, and traumatic injury, have variable clinical manifestations, and PNI has a high prevalence of 8% (nontraumatic cases)^2^ and 5% (traumatic cases).^3^ Neuron damage and end‐organ denervation can be corrected by the collateral branching of intact axons and the regeneration of injured axons. However, the progress of axon regrowth is relatively slow, and it may not always be possible to compensate for the dystrophic changes and irreversible atrophies of target tissues. In such cases, the end organ eventually undergoes degeneration, thus resulting in poor functional outcome and prognosis.^4^, ^5^ Therefore, how to promote nerve repair in the peripheral nerve system (PNS) has become a popular subject of interest, and researchers have developed many strategies for this. Autologous nerve grafts, synthetic conduits, stem cell transplantation, and secreted extracellular vesicles are the therapies that are currently popular.^6^ In particular, stem cell therapy and exosome therapy have changed the face of therapeutic treatments for many diseases,^7^, ^8^ and these therapies hold immense potential for the treatment of PNI.

Schwann cells (SCs) initiate proliferation and regulate many aspects of axonal regeneration in the distal nerve stumps after PNI. Schwann cells have versatile functions, including the production of neurotrophic factors, organization of myelin breakdown, promotion of axon growth, and re‐direction of axons to their targets; thus, the PNS has immense regeneration potential.^9^, ^10^, ^11^ Damage or death of SCs induced by injuries adversely affects nerve regeneration, and therefore, ensuring the survival of SCs is particularly important for peripheral nerve repair. In this regard, exogenous stem cells, especially adipose‐derived stem cells (ADSCs), have a unique advantage, as they are able to differentiate into SCs.^12^ Further, they are easily accessible^13^ and can induce upregulation of myelin‐associated markers.^14^ To date, ADSCs have shown the greatest potential, among other stem cells, to promote axonal regeneration.

Exosomes are secreted extracellular vesicles that play a pivotal role in mediating intercellular communication,^15^, ^16^ and they have reported to have therapeutic potential in PNS regeneration.^17^ SC‐derived exosomes can be internalized by axons to enhance regeneration in vivo.^18^ Specifically, ADSC‐derived exosomes (ADSC‐Exo) have the ability to accelerate cutaneous wound healing by overexpression of Nrf2,^19^, ^20^ and mRNAs and miRNAs contained within ADSC‐Exo promote neurite outgrowth.^21^ Our previous studies showed that ADSCs‐Exo could ameliorate SCs apoptosis after sciatic nerve injury of rats in vivo.^22^ However, the mechanisms underlying the interactions between ADSCs and SCs have not been elucidated, and the effects of ADSCs and exosomes on SC survival have not been studied yet.

In the present study, we have examined the therapeutic effect of ADSC treatments in PNI. Further, we have explored the effects of ADSC‐Exo transplantation on SC proliferation and the repair of peripheral nerves. The findings indicate that ADSCs and ADSC‐Exo therapy may have important implications in the treatment of PNI.

## 2 METHODS

### 2.1 Animal model

For all the experiments, male Wistar rats (age, 6 weeks; weight, 220‐240 g) were obtained from the Experimental Animal Center of the Second Military Medical University. All the animal experiments met the requirements of the Ethics Committee. In order to create the PNI model, the animals were first anesthetized with intraperitoneal administration of chloral hydrate (3.5 mL/kg), and a skin incision was made on the lateral aspect of the mid‐thigh of the left hind limb. Then, the left biceps femoris and the gluteus superficialis were separated by blunt dissection and the sciatic nerve was exposed. Sciatic nerve injury (SNI) was induced by crushing the nerve three times (5 seconds each time, with an interval of 10 seconds [fixed time]) at a position approximately 6‐8 mm distal from the ischial tuberosity (fixed position). A pair of hemostatic forceps (26 cm, Q211.26) was used with a force of 5 kg (fixed pressure). The injury site was measured within a 3 mm diameter from the point of application. In a separate group of sham‐operated mice, the left sciatic nerve was exposed without inducing the injury. For 30 minutes after suturing of the wound, motor nerve conduction velocity (MCV) was measured. An MCV of 10 m/s was indicated that the SNI animal model had been successfully established. All animals were housed in temperature‐ and humidity‐controlled large cages and allowed free access to water and food.

### 2.2 Isolation and culture of ADSCs

Adipose tissue was harvested from normal Wistar rats. Adipose‐derived stem cells were carefully isolated from rat inguinal fat pads, dissected, and minced using sterile scissors under aseptic conditions. Subsequently, the tissue was mechanically chopped with intermittent shaking and enzymatically dissociated at 37°C for 60 minutes with 0.1% collagenase I (Sigma C0130). The dissociated cells were cultured in RPMI‐1640 medium (Hyclone, SH30809.01B) containing l‐glutamine for 24 hours at 37°C in a 5% CO_2_ atmosphere. Nonadherent cells were removed on replacement of the medium every 2‐3 days, when the cells reached 80% confluence. Cultures were maintained at subconfluent levels in a 37°C incubator with 5% CO_2_ and passaged with trypsin/EDTA when required.

### 2.3 Characterization of ADSC surface molecules using flow cytometry

Rat ADSCs at the third passage were harvested by trypsinization, fixed in neutralized 4% paraformaldehyde for 30 minutes at a density of 1 × 10^6^ cells/mL, and incubated at 4°C overnight in the dark with the following antibodies: CD29 (1:100; BD, No‐610467), CD34 (1:100; Abcam, No‐ab81289), CD44 (1:400; Novus, No‐AF6127SP), CD45 (1:100; Novus, No‐NB100‐64895SS), and CD90 (1:100; BioLegend, No‐202501). Cells were washed in 1 mL PBS and centrifuged at 200 *g* for 10 minutes. Flow cytometry analysis was used to calculate the percentage of immunopositive cells.

### 2.4 Isolation and identification of SCs

At postsurgery 24 hours, SCs were isolated from an area 2 cm distal to the injury site of the sciatic nerve. In the sham‐operated group, SCs were isolated from… The perineurium was extracted and dissected into 1‐mm^3^ fragments. The explants were dissociated with 0.05% collagenase for 30 minutes and treated with 0.25% trypsin for 5 minutes at 37°C. Cells were plated on laminin (40 ng/mL) in Dulbecco's modified Eagle medium (DMEM, Invitrogen) supplemented with 10% fetal bovine serum (FBS, Invitrogen) and 10% penicillin‐streptomycin (Invitrogen). Fibroblasts were eliminated by the differential adhesion method, and SCs were isolated.^22^

Third‐passage SCs were plated on 24‐well plates and incubated for 24 hours at 37°C. The Cell Counting Kit‐8 (CCK8) assay was used to examine the proliferation of SCs at 0, 24, 48, 72, and 96 hours. Fluorescent secondary antibodies against myelin protein zero (MPZ) (1:500; PTZ, No‐10572‐1‐AP) and DAPI were added and incubated at room temperature for 30 minutes. Slides were examined using the Leica fluorescence microscope (DM6000B), and the number of immunopositive cells was counted to assess the nerve injury.

### 2.5 Isolation and characterization of ADSC‐derived exosomes

At passage 3, subconfluent ADSC cultures in the log phase were supplemented with 10% exosome‐free FBS, which was obtained by filtering the supernatants through 0.22‐μm pore filters (Millipore) and ultracentrifuging the serum at 100 000 *g* for 18 hours. We collected the ADSCs in the conditioned medium and isolated the exosomes according to the protocol of the Exosomal Protein Extraction kit (Wako MagCapture Exosome, No‐293‐77601). The protein concentration of the exosomes was examined by using the Pierce™ ECL Western Blotting Substrate (Thermo Fisher Scientific) and BCA Protein Assay Kit (Thermo Scientific), and for particle size determination, nanoparticle tracking analysis (NTA) was performed.^23^, ^24^

### 2.6 Transwell coculture of ADSCs and SCs

Adipose‐derived stem cells and SCs were prepared as previously described. The cells were divided into three groups: Sham‐SCs, SNI‐SCs, and SNI‐SCs + ADSCs. The Sham‐SCs and SNI‐SCs were harvested as previously described and cultured in 6‐well plates. In the SNI‐SCs + ADSCs group, to verify the effect of soluble factors secreted by ADSCs on SC proliferation, ADSCs and SCs were cocultured using a Transwell system to avoid intercellular contacts with the 0.4‐μm porous membrane. First, 0.5 mL of the SC suspension with a concentration of 1 × 10^6^ cells/mL was added in the lower wells in 1.5 mL of DMEM/F12 medium (10% FBS), and 0.5 mL of the ADSC suspension was plated in the upper wells. The concentration of ADSCs was adjusted to 5 × 10^5^ cells/mL. Cultures were maintained at subconfluent levels in a 37°C incubator in a 5% CO_2_ atmosphere, and the CCK8 assay was used to examine the proliferation of SCs at 0, 24, 48, 72, and 96 hours. After 24 hours of coculturing, we separated the chambers and determined the apoptosis rate of SCs by Annexin V/propidium iodide (PI) staining. qRT‐PCR was performed to analyze the expression of Bcl‐2 and Bax, which are apoptosis‐related proteins.^25^

### 2.7 Coculture of ADSC‐Exo and SCs

ADSC‐Exo were isolated as previously described. Schwann cells from injured nerves were first plated and grown to an appropriate density in the cell plate. Then, ADSC‐Exo were adjusted to the concentration of 0 μg/mL, 6.25 μg/mL, 12.50 μg/mL, 25.00 μg/mL, and 50.00 μg/mL and added to the culture. Accordingly, there were six groups of cells: Sham‐SCs, SNI‐SCs, SNI‐SCs + ADSC‐Exo (6.25 μg/mL), SNI‐SCs + ADSC‐Exo (12.50 μg/mL), SNI‐SCs + ADSC‐Exo (25.00 μg/mL), and SNI‐SCs + ADSC‐Exo (50.00 μg/mL). Flow cytometry was used to determine the rate of apoptosis of SCs, and the CCK8 assay was also performed at 0, 24, 48, 72, and 96 hours. Moreover, qRT‐PCR was performed to analyze Bcl‐2 and Bax expressions.

### 2.8 Statistical analysis

Animals were randomly assigned to each group. To determine the statistical significance of the difference between the mean ± standard deviation (SD) values of the datasets, repeated measurement analysis of variance (RMANOVA) was performed using the SPSS19.0 software. Statistical significance was set at *P* < 0.05 and *P* < 0.01.

## 3 RESULTS

### 3.1 Decrease in proliferation and increase in apoptosis of SCs after SNI

**Figure 1:**
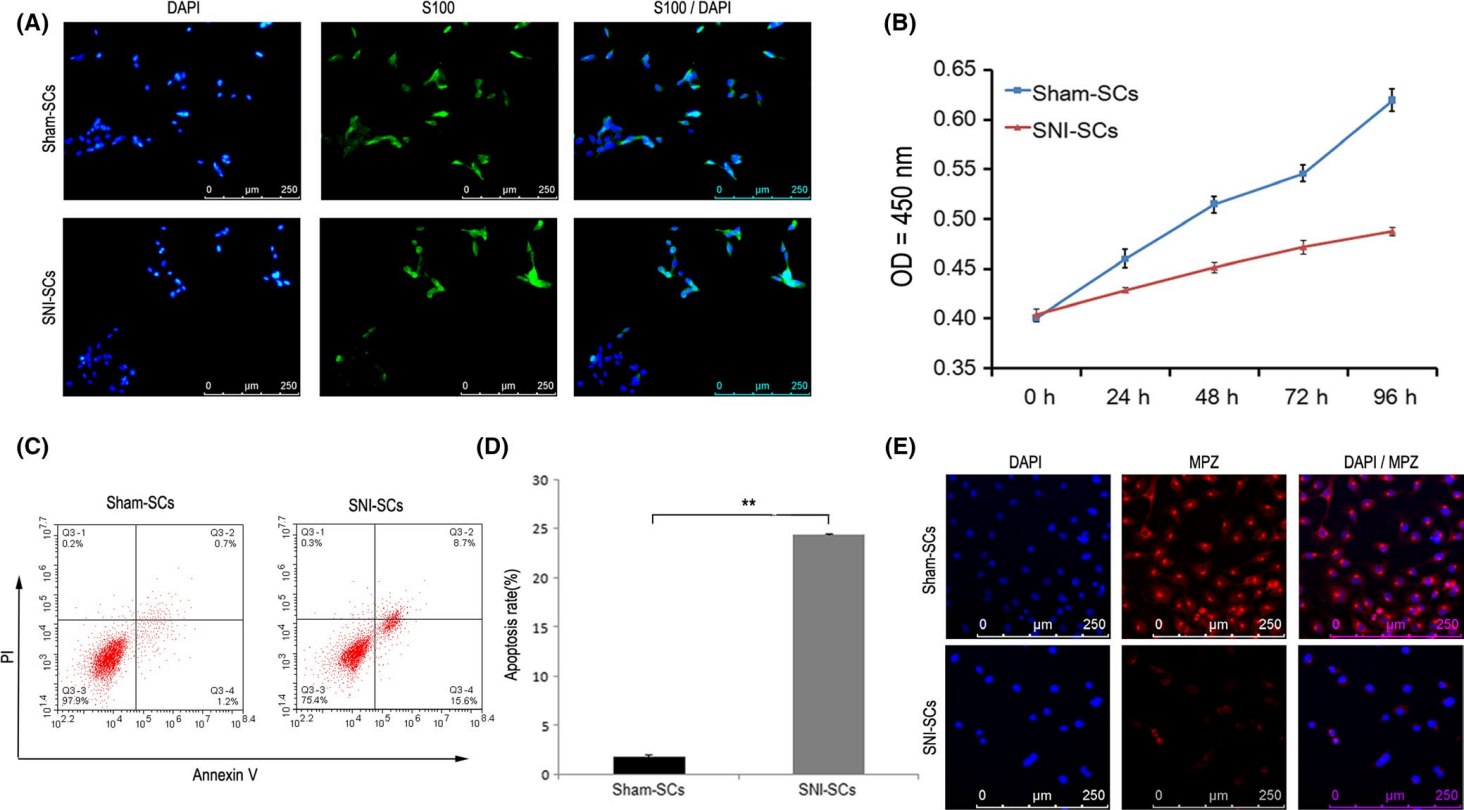
Decrease in SC proliferation and increase in SC apoptosis following sciatic nerve injury. A, Immunofluorescence staining showed that Sham‐SCs and SNI‐SCs express the SC marker S100. Scale bar: 20 µm. B, The cell proliferation curve of Sham‐SCs and SNI‐SCs, according to the CCK8 assay findings. Repeated measurement variance analysis, *F* = 254.067, *P* < 0.001. C, Apoptosis of Sham‐SCs and SNI‐SCs, as demonstrated by Annexin V and PI staining analysis. D, The apoptosis rate of SNI‐SCs was significantly increased as compared to Sham‐SCs. Data represent the mean ± SD values. **P < 0.01, Student's *t* test. E, Immunofluorescence staining showed that the expression of MPZ was lower in SNI‐SCs than in Sham‐SCs. Scale bar: 20 µm

Schwann cells isolated from the SNI and sham mice were mostly long and spindle‐shaped and expressed S100. However, some of the SNI‐SCs had a nearly round shape (Figure 1A). The CCK8 assay demonstrated that the proliferation rate of SNI‐SCs was decreased (Figure 1B). Annexin V‐FITC‐PI flow cytometry analysis showed that the apoptosis rate of SNI‐SCs was higher than that of Sham‐SCs (Figure 1C,D). Fluorescence microscopy analysis revealed that the number of SCs labeled by MPZ and DAPI was decreased after injury (Figure 1E). These findings imply that SNI resulted in increased apoptosis and decreased proliferation of SCs.

### 3.2 Reversal of the effects of SNI after coculture of SCs with ADSCs

**Figure 2:**
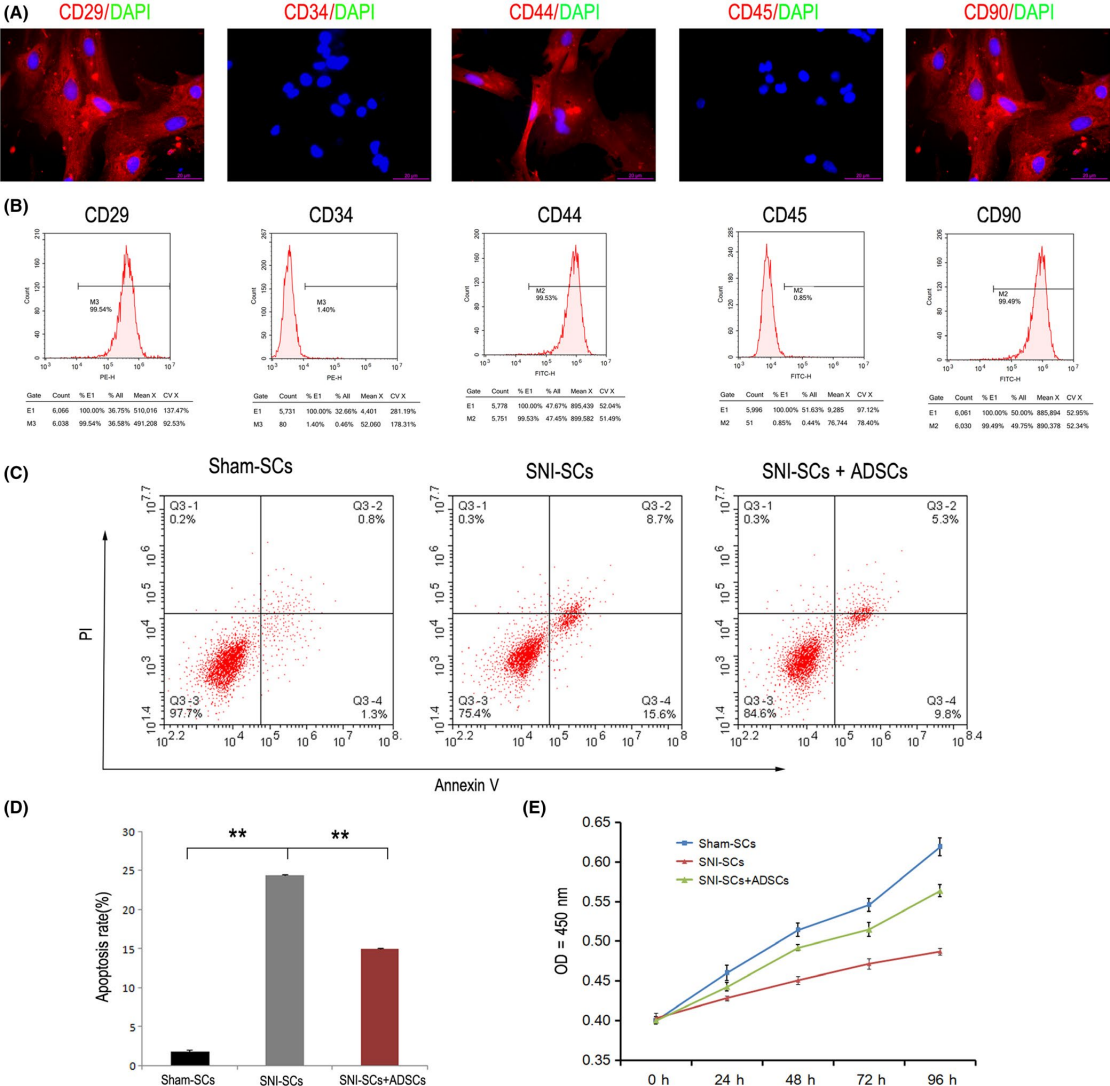
Decrease in SC apoptosis and increase in SC proliferation after coculture with ADSCs. A, Immunofluorescence staining showed that ADSCs expressed CD29, CD44, and CD90, but they did not express CD34 and CD45. Scale bar: 20 µm. B, Flow cytometry analysis of apoptosis. C, Apoptosis of Sham‐SCs, SNI‐SCs, and SNI‐SCs + ADSCs was analyzed by Annexin V and PI staining. D, The apoptosis rate of SNI‐SCs decreased significantly after treatment with ADSCs. Data represent the mean ± SD values. **P < 0.01, one‐way ANOVA. E, The cell proliferation curve of Sham‐SCs, SNI‐SCs, and SNI‐SCs + ADSCs, as measured by the CCK8 assay. Repeated measurement variance analysis, *F* = 204.983, *P* < 0.001. LSD *t* test, *P* < 0.05

Immunofluorescence staining showed that cultured ADSCs were positive for CD29, CD44, and CD90, but they were not positive for CD34 and CD45 (Figure 2A). CD29^+^CD44^+^CD90^+^CD34^−^CD45^−^ ADSCs were also detected by flow cytometry (Figure 2B). Flow cytometry also showed that coculture of ADSCs with SCs led to a decrease in the rate of SNI‐induced apoptosis (Figure 2C,D). Further, the CCK8 analysis showed that coculture with ADSCs also increased the proliferation rate of the injured SCs (Figure 2E). Thus, ADSC treatment seems to promote SC proliferation and ameliorate SC apoptosis after SNI.

### 3.3 Amelioration of the effects of SNI on SCs by ADSC‐Exo treatment

**Figure 3:**
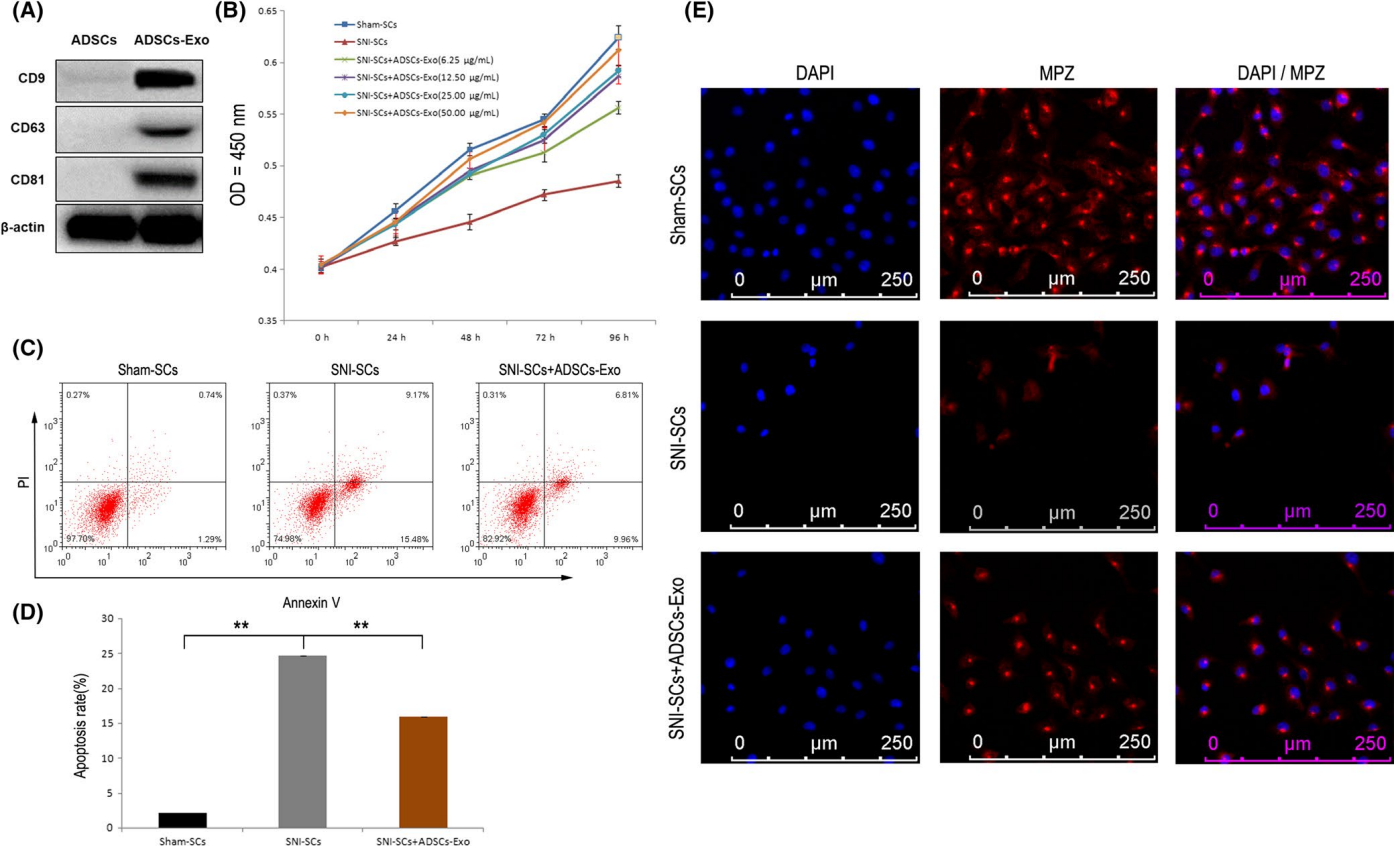
Increase in SC proliferation and decrease in SC apoptosis with ADSC‐Exo treatment. A, Western blot analysis showed that ADSC‐Exo expressed CD9, CD63, and CD81, but ADSCs did not express these proteins. B, The cell proliferation curve of SNI‐SCs treated with different concentrations of ADSC‐Exo. Repeated measurement variance analysis, *F* = 19.2, *P* < 0.001. LSD *t* test, *P* < 0.05. C, Apoptosis of Sham‐SCs, SNI‐SCs, and SNI‐SCs + Exo was analyzed by Annexin V and PI staining. D, The apoptosis rate of SNI‐SCs decreased significantly after treatment with ADSC‐Exo. Data represent the mean ± SD values. **P < 0.01, one‐way ANOVA. E, Immunofluorescence staining showed that the expression of MPZ was increased in SNI‐SCs treated with ADSC‐Exo. Scale bar: 20 µm

Exosomes were isolated and purified from ADSCs using the Exosome Isolation Kit, and they were termed ADSC‐Exo. The BCA method was performed to quantify the protein concentration of the isolated exosomes. The protein concentration of the extracted exosomes was determined to be 1.44 μg/mL. Nanoparticle tracking analysis revealed that the isolated sample contained nanovesicles (40‐100 nm), and the ADSC‐Exo had diameters of 40‐60 nm (1.22%), 60‐80 nm (30.7%) and 80‐100 nm (68.1%). Western blot analysis showed that the vesicles expressed CD9, CD63, and CD81, which are not expressed by ADSCs (Figure 3A).

To determine the effect of ADSC‐Exo on the proliferative kinetics of SCs, we examined the proliferation rate of SCs after treatment with ADSC‐Exo at concentrations of 6.25 μg/mL, 12.5 μg/mL, 25.0 μg/mL, and 50.0 μg/mL. The CCK8 assay showed that the proliferation rate of the treated SCs increased significantly in a time‐ and concentration‐dependent manner. The increase in the proliferation rate of SNI‐SCs treated with 50.0 mg/mL ADSC‐Exo for 96 hours was most pronounced at 50.5 (Figure 3B). Therefore, 50.0 μg/mL exosome treatment for 96 hours was used for subsequent experimental analysis.

Flow cytometry analysis showed that the ADSC‐Exo treatment resulted in a significant decrease in the percentage of apoptotic cells (*P *< 0.01; Figure 3C,D). Immunofluorescence staining of MPZ and DAPI showed that the number of apoptotic cells was significantly reduced when injured SCs were cocultured with ADSC‐Exo (Figure 3E). These findings indicate that ADSC‐Exo have an anti‐apoptotic effect in injured SCs, and also promote the proliferation of SCs.

### 3.4 Effect of ADSCs and ADSC‐Exo on the expression of Bax and Bcl‐2 in SCs

**Figure 4:**
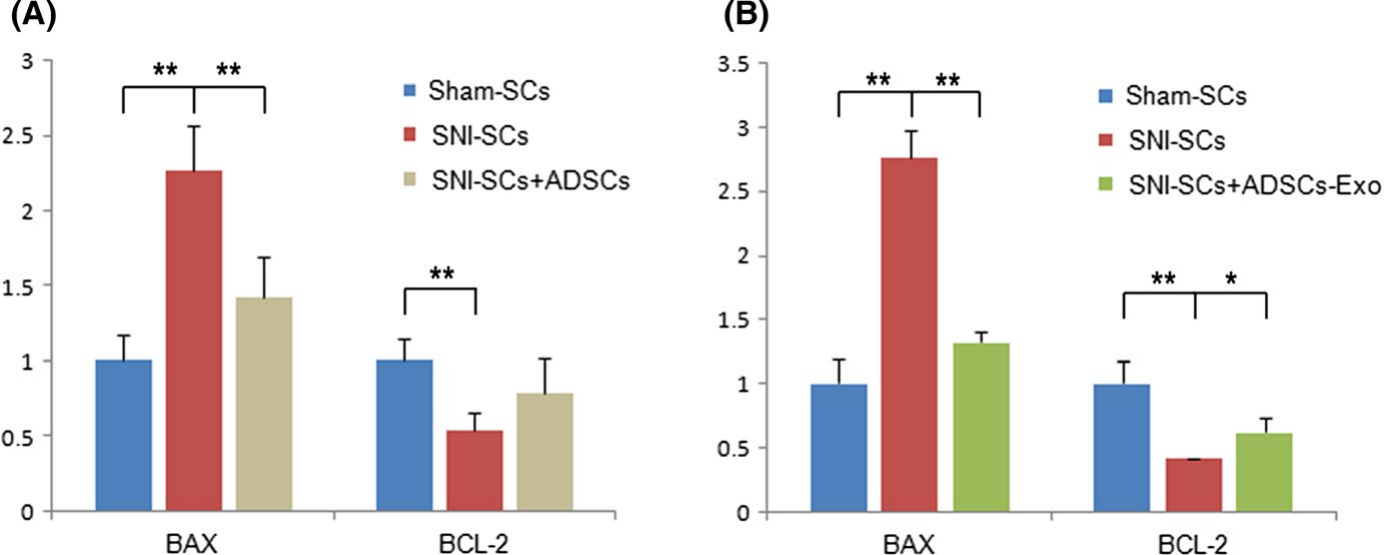
Downregulation of Bax expression and upregulation of Bcl‐2 expression in SNI‐SCs. A, qPCR analysis showed significantly downregulated expression of Bax and significantly upregulated expression of Bcl‐2 with ADSC treatment. B, qPCR analysis showed significantly downregulated expression of Bax and significantly upregulated expression of Bcl‐2 with ADSC‐Exo treatment. Data represent the mean ± SD values. **P < 0.01, *P < 0.05, one‐way ANOVA

Bax and Bcl‐2 are members of the Bcl‐2 gene family. Bcl‐2 is an anti‐apoptotic factor, while Bax is a pro‐apoptotic factor.^6^ qRT‐PCR analysis showed that the mRNA expression of Bcl‐2 decreased and that of Bax increased significantly in the SNI‐SCs. After coculture with ADSC‐Exo, the expression of Bcl‐2 increased and that of Bax decreased (Figure 4A), and ADSC‐Exo treatment had similar effects (Figure 4B). The results indicate that ADSCs and ADSC‐Exo affected the apoptosis and proliferation of SCs through a regulatory effect on apoptosis‐related genes.

## 4 DISCUSSION

In the current study, we utilized the nerve crush model to mimic axonotmesis,^26^ in which the connective sheath and SCs contained in basal lamina tubes remain intact but axons are disrupted. In this model of PNI, the packed myelin sheath at first loosens up and breaks down into fragments within 24‐48 hours following nerve injury.^27^ This is followed by demyelination that involves both SC reprogramming‐mediated myelinophagy and macrophage‐mediated phagocytosis.^28^, ^29^, ^30^ During the recovery process, the distal end undergoes Wallerian degeneration, and SCs proliferate to form Büngner bands that guide axonal outgrowth.^10^, ^31^ Furthermore, recently, researchers have found that nerve injury triggers the conversion of myelin and nonmyelin (Remak) SCs to a cell phenotype that is specialized for repair,^32^ and SCs undergo reprogramming to acquire an invasive mesenchymal‐like cell phenotype in the wound microenvironment in order to drive PNS regeneration.^33^ Thus, SCs play a complex role in peripheral nerve regeneration, which is heavily dependent on the presence of living SCs and their multiple functions. Based on these findings, we concluded that in the present axonotmesis model, SCs are the major components of the PNS that undergo apoptosis.

Emerging evidence has shown the therapeutic roles of exosomes from ADSCs with regard to tissue repair and cell‐free therapy. Studies have shown that ADSC‐derived exosomes can bring about significant restoration of cavernous nerve injury in rat models.^34^ Moreover, they were found to have protective roles in ischemic stroke^35^ and wound healing.^36^ These beneficial effects of ADSC‐derived exosomes go beyond the known therapeutic applications of exosomes in RNA interference therapy and drug delivery systems and as disease biomarkers.^37^ Based on these findings, in the present study, we hypothesized that exosomes from ADSCs inhibit the apoptotic process of SCs and promote their proliferation in rat models of SNI. This hypothesis was proven through in vitro experiments on SCs after SNI in the present study. Further, our experiments demonstrated that ADSC‐derived exosomes reduced the apoptosis rate in injured peripheral nerves by upregulating the anti‐apoptotic Bcl‐2 mRNA expression and downregulating the pro‐apoptotic Bax mRNA expression, and also promoted the proliferation of SCs. Hence, the present study demonstrates that exosomes from ADSCs exert neuroprotective effects on SCs in the SNI model.

In addition to the beneficial effects of ADSCs, the effects of ADSC‐secreted cytokines on SC proliferation and the self‐influence of SCs on proliferation should also be considered. In addition, we cannot rule out the programmed cell death of SCs that occurs during the physiological growth of peripheral nerves, wherein excess SCs without axonal contact are removed by apoptosis to eliminate supernumerary SCs during the proliferative period.^27^, ^38^, ^39^ Further, autophagy of SCs is initiated for the clearance of myelin after neuronal damage in order to generate a favorable environment for axonal regrowth.^36^ Intriguingly, MLKL, which is known to rupture the cell membrane during necroptotic cell death, has been reported to target SCs to promote myelin breakdown and axon regeneration.^29^ Thus, the apoptosis of SCs is a complex and multifaceted phenomenon in peripheral nerve repair. Nonetheless, our findings delineate a novel exosome‐mediated mechanism for ADSC‐SC cross talk that reduces apoptosis and promotes the proliferation of SCs. Further investigations into this mechanism can help establish potential PNI therapies.

## 5 CONCLUSION

In conclusion, our findings demonstrate that ADSC‐derived exosomes promote PNS regeneration by reducing apoptosis and promoting the proliferation of SCs through upregulation of Bcl‐2 mRNA expression and downregulation of Bax mRNA expression. Thus, we provide an experimental basis for the clinical application of exosomes from ADSCs to promote regeneration following PNI.

## CONFLICT OF INTEREST

The authors declare no conflict of interest.
