#  

**DOI:** 10.1111/cns.13833

**Published:** 2022-04-05

In Lin et al.,^1^ the immunofluorescence of CD29 and CD90 are exactly the same in Figure 2A. The correct Figure 2A is given below:

**FIGURE 2:**
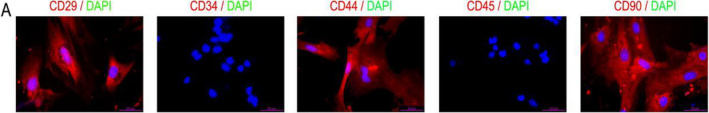
Decrease in SC apoptosis and increase in SC proliferation after coculture with ADSCs. (A) Immunofluorescence staining showed that ADSCs expressed CD29, CD44, and CD90, but they did not express CD34 and CD45. Scale bar: 20 μm

The authors apologize for the error.
